# Notes on Preparations for the Sick

**Published:** 1896-12

**Authors:** 


### Notes on Preparations for the Sick.

**Carniferrine.**—Lucius & Brüning, Hoechst on the Maine.—This powder, which can be obtained through the Jeyes' Sanitary Compounds Co., contains thirty per cent. of iron in organic combination, being an iron salt of phospho-carnic acid. It is prepared from extract of meat. The dose is eight grains per day, given in powder with sugar. We cannot have too many iron preparations. This is one of the many which are quite worthy of trial. As a rule we prefer the inorganic iron preparations, but they do not always answer our expectations, when the organic compounds sometimes do better.

**Antikamnia.**—The Antikamnia Chemical Co., St. Louis.—This preparation, for which Mr. John M. Richards is the British agent, is sent out in the form of powder or tablet. For several years this drug has been more or less in use for the relief of pain, but has not so firmly established itself in this country as in the home of its manufacture. It is described as a combination of coal tar derivatives of the series $C_n H_{2n-6}$, into which the amines have entered, forming the various amido-compounds. The white, alkaline powder is moderately soluble in water, but freely so in spirits. The five-grain tablet is a convenient mode of administration, three of them giving a full dose for adults. They appear to give relief to neuralgic pains, and have a sedative effect on the nervous system when over-excited.

**Pumiline Zalones.**—G. & G. Stern, London.—**Zalones** are a variety of the *genus* lozenge containing a basis of chlorate of potash, milk sugar, gum arabic and liquorice. Each contains one minim of Pumiline essence and one minim of menthol. It is a pleasant, and should prove to be a very useful lozenge, in spite of its somewhat heathenish designation, which even the promoters spell differently, as in one place we find them called "Zablones."

**Peptonised Cocoa and Milk. Café Zylak.**—Savory & Moore, London.—The well-known and most useful preparation of peptonised milk and cocoa is much appreciated by those who do not dislike sweet things and have no objection to cocoa. A similar preparation with coffee instead of cocoa must meet with a large demand. Where peptonised milk is needful these two preparations give us an opportunity of a variation in the diet which is much appreciated, and the difficulties of peptonisation are escaped. The coffee preparation is already giving much satisfaction.

---

**Maltine with Coca Wine.**—The Maltine Manufacturing Company Ltd., London.—Each fluid ounce of this viscid luscious fluid contains the active ingredients of thirty grains of coca leaves. It must therefore combine the invigorating offices of the coca with the digestive and nutrient qualities of a good preparation of maltine.

---

**Neo-Kola.**—Thomas Christy & Co., London.—New beverages are always in demand, and this one has many special features. It is unmixed with any foreign substance beyond a mere trace of vanilla flavouring. It contains appreciable quantities of caffeine and theobromine with kolanin. It makes an excellent beverage, and is less likely to disturb the digestion than the ordinary kinds of tea, coffee, or cocoa. For the tired brain-worker it is likely to be a very efficient restorative.

---

**Nutrient Suppositories.**—Parke, Davis & Co., London.—Each of these excellently made suppositories contains about 125 grains of lean beef, almost entirely peptonised. We have a decided preference for fluid enemata, but in cases where the fluids are not retained, or will not be tolerated from any cause, the nutrient in suppository form becomes essential. Rectal food should obviously be pre-peptonised, and these suppositories provide a soluble form of peptone, which should in due time after solution become readily absorbed.

---

**Soloids: Carbolic Acid; Corrosive Sublimate; Potassium Permanganate; Silver Nitrate; Zinc Chloride.**—Burroughs, Wellcome & Co., London.—If any of our readers are unaware of the existence of these useful preparations, it will be merely necessary for us to call attention to them, in order that their advantages might be at once appreciated. The convenience of being able with so much ease to prepare lotions of definite strength will be at once apparent. Any risk of confounding them with the better known tabloids is obviated by the difference in shape. Each of the **Carbolic Acid** soloids contains exactly five grains, and when dissolved in five ounces of water makes a solution suitable for use as a disinfectant or antiseptic spray.

The strength of the others is as follows: **Corrosive Sublimate**, 1.75 grains; **Potassium Permanganate**, five grains; **Silver Nitrate**, one grain; **Zinc Chloride**, one grain.

---

**Dr. Balme's Sublimated Paper.**—B. Kühn, London.—Each leaf of this **Paper** contains eight grains of perchloride of mercury, and will rapidly dissolve in water. One leaf in a quart of water makes a lotion of 1 : 2500.

---

**Taka-Diastase.**—With reference to the notice on page 277 in our last number, it has been pointed out that the ferment will do more than we implied, for it is capable of converting over one hundred times its weight of starch into sugar, *in a glass vessel in ten minutes, with the products of its conversion hampering its action.* In three hours it will convert over one thousand times its own weight. The powder appears to be the fungus itself, the Eurotium oryzæ, which is grown on wheat bran.

The compressed tablets, each containing 2½ grains, form a sufficient and convenient dose; they should be taken at the commencement of a meal in order that the diastasic action may come into play before the food becomes saturated with acid gastric juice.

